# Statement Recognition of Access Control Policies in IoT Networks

**DOI:** 10.3390/s23187935

**Published:** 2023-09-16

**Authors:** Li Ma, Zexian Yang, Zhaoxiong Bu, Qidi Lao, Wenyin Yang

**Affiliations:** School of Electronic Information Engineering, Foshan University, Foshan 528000, China2112151176@stu.fosu.edu.cn (Z.Y.);

**Keywords:** access control policies, IoT networks, federated learning, pre-trained word embeddings, access control policy recognition, data privacy, IoT applications

## Abstract

Access Control Policies (ACPs) are essential for ensuring secure and authorized access to resources in IoT networks. Recognizing these policies involves identifying relevant statements within project documents expressed in natural language. While current research focuses on improving recognition accuracy through algorithm enhancements, the challenge of limited labeled data from individual clients is often overlooked, which impedes the training of highly accurate models. To address this issue and harness the potential of IoT networks, this paper presents FL-Bert-BiLSTM, a novel model that combines federated learning and pre-trained word embedding techniques for access control policy recognition. By leveraging the capabilities of IoT networks, the proposed model enables real-time and distributed training on IoT devices, effectively mitigating the scarcity of labeled data and enhancing accessibility for IoT applications. Additionally, the model incorporates pre-trained word embeddings to leverage the semantic information embedded in textual data, resulting in improved accuracy for access control policy recognition. Experimental results substantiate that the proposed model not only enhances accuracy and generalization capability but also preserves data privacy, making it well-suited for secure and efficient access control in IoT networks.

## 1. Introduction

Ensuring secure and authorized access to resources is paramount in IoT networks. IoT networks are increasingly susceptible to a variety of attacks [1,2], making data and privacy protection of utmost importance. Among various security services, access control [3] plays a pivotal role in managing and restricting resource access, ensuring that only authorized users or entities can access specific resources or perform specific operations, thus preventing unauthorized access, data leakage, and information tampering. Therefore, access control is crucial for safeguarding the security of IoT applications [4].

In IoT access control [5,6,7], Access Control Policies (ACPs) [3,8] are pivotal in determining the authorization or non-authorization of resource access. These policies are typically described in natural language within project specification documents of organizational entities [9]. The task of recognizing access control policies involves identifying statements related to access control from project documents. Traditional ACP recognition relies on manual filtering [10]. In recent years, machine learning and deep learning techniques have been applied to enhance ACP recognition; however, current solutions [11] often suffer from low accuracy and data privacy concerns. This challenge is particularly pronounced in IoT access control [12], where improving the accuracy of ACP recognition while ensuring the security and privacy of user data is a significant challenge.

Upon analysis, the shortage of publicly available labeled data is a critical factor affecting the accuracy of training ACP recognition models. The key lies in how to effectively utilize access control policies from different data sources for collaborative model training while preserving the privacy of ACP data. Federated Learning (FL) [13,14,15,16] provides an effective approach to collaboratively train models while protecting data privacy. By adopting FL, a collaborative training model can be built using ACPs from various sources without the need for centralized data computation, addressing the issues of low accuracy and a lack of labeled data in ACP recognition models.

This paper proposes a framework named FL-Bert-BiLSTM for ACP recognition. Experimental results demonstrate that the proposed framework achieves high-precision ACP recognition while preserving the privacy of ACP data. The main contributions of this paper are as follows:

- Introduction of federated learning into ACP recognition technology, constructing a privacy-preserving authorized ACP recognition framework.
- Enhancement of ACP recognition capability in a distributed environment by incorporating pre-trained word embeddings from Natural Language Processing (NLP) [17] into federated learning.
- Experimental results validate the effectiveness of the proposed model in significantly improving the accuracy of authorized ACP recognition while ensuring data privacy and security.

The findings of this research provide a novel perspective and approach to ACP recognition, offering an effective solution for practical applications. The subsequent sections are organized as follows: Section 2 introduces related work on ACP recognition implementation. Section 3 presents the methodology of the current research, including model architecture and algorithm analysis. Section 4 presents the results and analysis. Section 5 discusses the proposed research in the context of IoT networks. Finally, Section 6 concludes the paper.

## 2. Related Work

In earlier studies [18], access control policies were extracted from documents through manual analysis conducted by security experts or under Controlled Natural Language (CNL) conditions [11,19]. Although manual analysis tends to yield accurate results, it requires proficient security experts and a longer evaluation time. CNL, which aims to minimize ambiguity and complexity in natural language [19], can generate comprehensive results. However, CNL-based methods often rely on specialized generation tools to transform relevant vocabulary in the documents, resulting in lower flexibility and limitations in their application scenarios.

Modern studies have utilized natural language processing techniques to identify access control policies from documents. Table 1 presents a summary of the findings. Xiao et al. proposed a method called Text2Policy [20], which utilizes shallow parsing techniques and finite-state transducers to match sentences with one of four predefined access control patterns. Their approach achieved a recognition accuracy of 88.7%. However, it fails to capture access control policies that do not adhere to the pre-defined semantic patterns, with only 34.4% of such policies being captured [21].

Slankas introduced Access Control Rule Extraction (ACRE) [22], a machine learning-based method for identifying access control policies from natural language documents. The author investigated whether words, synonyms of words, part-of-speech tags, and named entities can serve as indicators for recognizing access control policy sentences. The proposed method achieved an accuracy of 87.3% when validated on the iTrust dataset consisting of 1159 sentences. Slankas et al. [21] extended the ACRE framework with minor modifications to its components and techniques. Unlike previous literature, they used a larger dataset of five policy documents to validate their proposed method. This supervised learning approach utilizes an ensemble classifier consisting of a k-nearest neighbors (k-NN) classifier, a naive Bayes classifier, and a support vector machine classifier. The method distinguishes access control policy sentences from other types of sentences by computing a threshold based on the ratio of the distance from the nearest neighbors to the number of words in the sentence. The average classification accuracy achieved was 81%. However, the k-NN classifier’s processing time is relatively slow due to the need for sentence comparison before making a decision.

Refs. [23,24] employ Semantic Role Labeling (SRL) to automatically identify the Predicate-Argument Structure (PAS) and extract access control policies from natural language requirement documents. A set of predefined rules is applied to the extracted arguments to define roles and construct a Role-Based Access Control (RBAC) system. Ref. [25] designs four types of features, namely, security features, PMI features, syntactic complexity features, and dependency features, to describe statements in the documents. Access control statement recognition is performed using a naive Bayes classifier and a Support Vector Machine (SVM). Ref. [26] utilizes a Recursive Neural Network (RNN) model to identify policy statements from natural language documents, but the overall performance is considered mediocre. Alohaly et al. [27,28] utilize a Convolutional Neural Network (CNN) to identify subject and object attributes related to system access control from natural language policy statements. However, this method is only for extracting attribute information related to access control. The related work on statement recognition of ACPs is given in Table 1.

**Table 1:** Statement recognition of ACPs in the related work.

Study	Underlying Tech.	Dataset	Performance
[20]	Semantic patterns matching	iTrust, IBM APP	Prec: 88.7%
[21]	K-NN	iTrust, IBM App, Cyberchair, collected ACP	Prec: 81%
[22]	K-NN, Naive Bayes, and SVM classifiers	iTrust	Prec: 87.3%
[25]	Naive Bayes and SVM classifiers	iTrust, IBM App, Cyberchair, collected ACP	Prec: 90%
[26]	Deep recurrent neural network	ACPData, iTrust, IBM App, Cyberchair, collected ACP	Prec: 81.28%

While the aforementioned studies primarily focus on improving the recognition of access control statements to enhance the recognition rate, they tend to overlook the sensitivity of data and the issues of data privacy and security, which are crucial in the era of big data. In contrast, federated learning relies on distributed training of models and gradient sharing across devices, serving as a privacy-enhancing approach [29,30]. This approach facilitates model training through collaborative sharing among multiple endpoints while preserving privacy.

However, many existing methods neglect the specific characteristics of IoT networks, which may lead to limited applicability in real-world IoT scenarios. In this research, we address these challenges and propose an FL-based framework, FL-Bert-BiLSTM, for privacy-preserving access control policy recognition in IoT networks. By leveraging federated learning and incorporating pre-trained word embeddings from Natural Language Processing (NLP), our approach achieves a balance between accuracy and privacy protection, making it well-suited for real-time and distributed training on edge devices in IoT networks.

## 3. The Proposed Method

**Figure 1:**
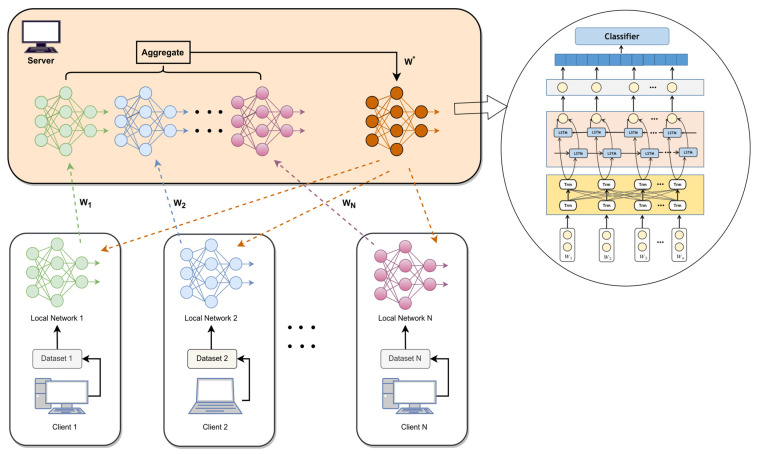
The deployment of an ACP identification model based on FL.

To improve the accuracy of Access Control Policy (ACP) recognition while ensuring data privacy, this paper proposes an ACP recognition technique called FL-Bert-BiLSTM. The deployment of the proposed FL-Bert-BiLSTM model is illustrated in Figure 1, and the following sections provide a detailed description of each component in these steps. Specifically, Bert encodes the ACP in NLACP along with their corresponding features. FL guarantees data privacy across client devices while expanding the data volume. Finally, through progressive data distillation, the features of the final sentence representation are transformed into predictive results.

### 3.1. Dataset Pre-Processing

The conversion of ACP statements into machine-readable vectors plays a crucial role in the recognition of access control policy statements. Traditional word embedding methods [31] can partially address the issue of contextual relationships between words, but they can only provide a fixed vector representation for each word and cannot resolve the problem of word ambiguity. By using dynamic word vector representation methods such as BERT to pre-process the data in this paper, the problem of word ambiguity can be better addressed, thereby enhancing the representational capacity and semantic understanding of access control policy statements. Additionally, dynamic word vectors can consider contextual information to better capture the meaning and purpose of access control policy statements, thereby improving the system’s security and stability. The obtained ACP statements in NLACP, denoted as $W_{1},W_{2},\ldots ,W_{n}$, are represented as the sum of word vectors, segment vectors, and position vectors, which serve as the input to the model. Multiple layers of bidirectional Transformer encoders, $T_{1},T_{2},\ldots ,T_{n}$ are obtained as the output feature vectors.

**Figure 2:**
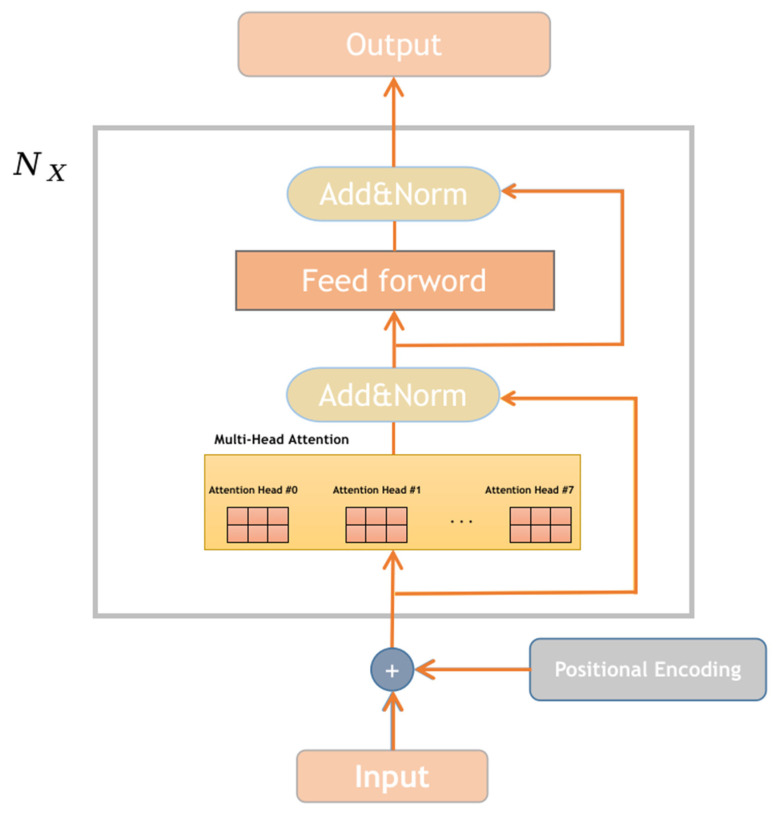
The structure of the transformer-encoder model.

Incorporating multiple bidirectional Transformer models, Bert [32] constitutes the core framework of the encoder section, enabling a more comprehensive capture of bidirectional relationships within NLACP, as illustrated in Figure 2, which displays the Transformer-encoder model structure. The encoder, denoted as $N_{x}$, is composed of N identical network layers, transforming access control policies into vectors by adding positional encodings to the input X. Subsequently, this output is multiplied by pre-trained weights to derive the $Q_{ACP}$, $K_{ACP}$, and $V_{ACP}$ matrices. The multiplication of the $Q_{ACP}$ and $K_{ACP}^{T}$ matrices computes the relevancy between individual words within the access control policy while preventing results from becoming overly large. This is achieved by dividing the product by the square root of $d_{k}$, where $d_{k}$ represents the vector dimensionality of $Q_{ACP}$ and $V_{ACP}$. The normalized relevancy scores between words in the access control policy are obtained using the Softmax function and are ultimately multiplied by $V_{ACP}$. This process yields new vector encodings for each word, facilitating the computation of inter-word weights within access control policy statements. Consequently, this series of calculations enables the determination of attention values [33] as depicted in Equation (1).

(1) $$A_{ttention}(Q_{ACP},K_{ACP},V_{ACP})=Soft\max(\frac{Q_{ACP}K_{ACP}^{T}}{\sqrt{d_{k}}})V_{ACP}$$

In the multi-head attention mechanism, to eliminate the influence of the initial values of $Q_{ACP}$, $K_{ACP}$, and $V_{ACP}$, h different weight matrices, $Q_{ACP}$, $K_{ACP}$, and $V_{ACP}$ are used for parallel computation. Finally, the different attention results are concatenated to obtain the multi-head attention values as shown in Equations (2) and (3).

(2) $$M_{ultiHead}(Q_{ACP},K_{ACP},V_{ACP})=C_{oncat}(h_{1},h_{2},\ldots ,h_{n})W^{o}$$

(3) $$h_{i}=A_{ttention}(Q_{ACP}W_{i}^{Q_{ACP}},K_{ACP}W_{i}^{K_{ACP}},V_{ACP}W_{i}^{V_{ACP}})$$

In the equations, $W_{i}^{Q_{ACP}}$, $W_{i}^{K_{ACP}}$, and $W_{i}^{V_{ACP}}$ represent the weight matrices of $Q_{ACP}$, $K_{ACP}$, and $V_{ACP}$ for the i-th head, respectively. $W^{o}$ represents an additional weight matrix, and $C_{oncat}(.)$ represents the concatenation function.

The obtained output vectors are then passed through residual connections and layer normalization layers, which are used to add the input and output of the current layer and perform normalization. Layer normalization transforms the inputs into mean and variance to increase non-linearity. Residual connections linearly combine the input X and the result F(X) obtained by applying a non-linear transformation to X and use the combined result as the output.

After obtaining the output from the residual and layer normalization, it is processed through a feed-forward neural network to generate the corresponding matrix $W_{e}$. This network layer consists of two fully connected layers, where one of the layers utilizes the ReLU activation function to enable more efficient computations and improve convergence speed.

(4) $$W_{e}=\max(0,XW_{1}+b_{1})W_{2}+b_{2}$$

In Equation (4), $W_{1}$ and $W_{2}$ represent the weight matrices of the two fully connected layers, while $b_{1}$ and $b_{2}$ represent the bias terms of the two fully connected layers.

Finally, the output results processed by the residual connections and layer normalization are obtained as the output of the encoder.

### 3.2. Bi-LSTM for ACP Identification

In access control text, there exists a correlation between words, including their context and sequential relationships. The traditional approach is to use LSTM to address the issue of long-term dependencies. LSTM is a variant of recurrent neural networks (RNNs) [34], which effectively tackles the problems of gradient explosion and poor ability to capture long-distance dependencies in traditional RNNs. It is more suitable for modeling time series data, such as text data. However, due to the limitation of utilizing only historical data, LSTM cannot leverage future data information. In other words, LSTM can only consider the previously encountered text content and cannot predict the influence of subsequent text content.

In this study, the optimized model BiLSTM, which is based on LSTM, is employed to capture the preceding and succeeding relationships in ACP. BiLSTM is a deep learning model that can be used for processing sequential data. It leverages forward LSTM and backward LSTM to process the forward and backward information of the input sequence, respectively, and then combines their outputs to obtain a sentence representation with global information. By using BiLSTM, it is possible to better capture the semantic relationships between words in a sentence, thereby achieving improved performance in access control text recognition tasks.

**Figure 3:**
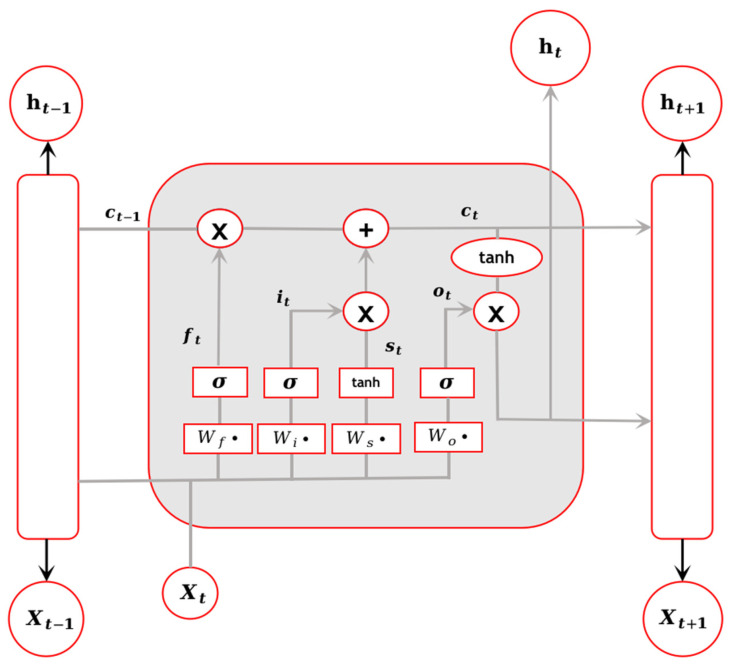
LSTM structure.

Unlike the traditional RNN sequential structure, LSTM focuses on the cell state, which interacts with the information carried by the cell state through “three gates”. In BiLSTM, there are four components: the input gate (i), the forget gate (f), the output gate (o), and the cell state (c). The specific structure of LSTM is illustrated in Figure 3.

By updating the LSTM network, the previous hidden state $h_{t-1}$ and the current input $X_{t}$ are obtained. The forget gate, controlled by a sigmoid layer, determines which information to forget from the cell state. The value of the forget gate $f_{t}$ is calculated as shown in Equation (5).

(5) $$f_{t}=\sigma (W_{f}\chi_{t}+\omega_{f}h_{t-1}+b_{f})$$

Next, we need to determine which information to store in the cell state. This is carried out by using the input gate $i_{t}$, controlled by a sigmoid layer, to decide the information that will be updated. Additionally, a new candidate value is created using a tanh layer. The values of the input gate and the temporary cell state $S_{t}$ are calculated as shown in Equations (6) and (7).

(6) $$i_{t}=\sigma (W_{i}\chi_{t}+\omega_{i}h_{t-1}+b_{i})$$

(7) $$S_{t}=\tanh(W_{s}\chi_{t}+\omega_{s}h_{t-1}+b_{s})$$

The current cell state $c_{t}$ is calculated by combining the input gate value $i_{t}$, the forget gate value $f_{t}$, and the temporary cell state $S_{t}$, as shown in Equation (8).

(8) $$c_{t}=f_{t}\times c_{t-1}+i_{t}\times S_{t}$$

A sigmoid output gate is established to determine which parts of the cell state will be outputted. The current hidden state $h_{t}$ at the current time step is determined by the combination of the output gate $o_{t}$ and the cell state $c_{t}$, as shown in Equations (9) and (10).

(9) $$o_{t}=\sigma (W_{o}\chi_{t}+\omega_{o}h_{t-1}+b_{o})$$

(10) $$h_{t}=o_{t}\times \tanh(c_{t})$$

In the equation, W and ω represent the weight matrices, and b represents the bias vector.

**Figure 4:**
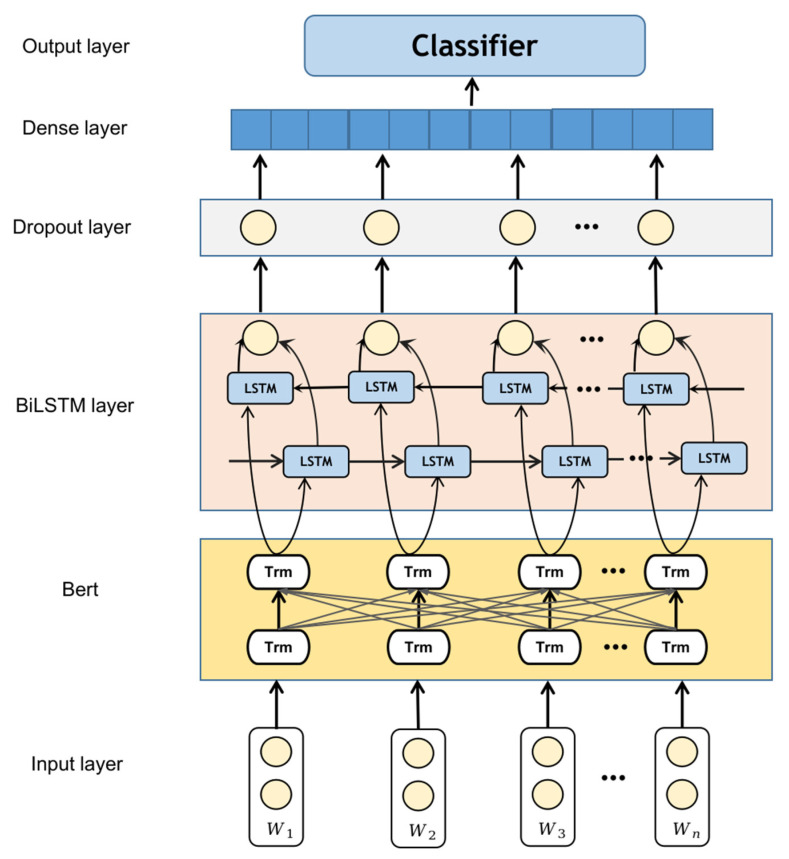
Flow of a Bert-BiLSTM model.

The context information in the opposite direction of the access control statement is captured by the hidden layer vectors outputted by the forward LSTM and backward LSTM units of the BiLSTM. The BiLSTM effectively utilizes the forward and backward feature information of the input. Figure 4 presents the BiLSTM algorithm framework designed for ACP recognition. The dropout layer is used to mitigate overfitting when there is a limited number of training samples. During training, some neurons are randomly deactivated, reducing the model’s complexity and preventing overfitting.

### 3.3. FL for ACP Identification

With the rise of issues such as spam emails and advertising harassment, people have become increasingly sensitive to privacy concerns. This is evident in laws like the General Data Protection Regulation (GDPR) implemented in Europe [35]. To a large extent, this will impact the maximization of data utilization. Therefore, for highly private ACP data that are predominantly stored on local clients, there is a need to utilize federated learning techniques [36,37] to obtain more ACP datasets for model training while ensuring the privacy and security of user data.

To address the issue of privacy in ACP recognition, the FL-Bert-BiLSTM architecture is constructed. In this architecture, assuming we have N clients, each client $C_{i}$ having its dataset $D_{i}$, the training process in FL is as follows: at the beginning of each communication round t, the central server distributes the global model $M_{t}$ to each client. Each client then loads the global model provided by the server and trains it using its dataset. The clients save the trained weights and upload them to the central server. Once all clients have completed training, the central server obtains the model weights from all clients and updates the global model weights using one of the popular algorithms, such as FedAvg (McMahan et al., 2017) [29], as shown in Formula (11):

(11) $$W_{t+1}\leftarrow \sum_{k=1}^{K}\frac{n_{k}}{n}W_{t}^{k}$$

where $n_{k}$ is the size of the dataset for each client, n is the total number of clients N, $W_{t}$ is the weight parameter of the client at time t, and $W_{t+1}$ is the aggregation of weight parameters received by the global server from all clients. After the computation, the weighted average of the new weight parameters is obtained. Then, the new weight parameters $W_{t+1}$ are loaded to obtain the new global model. Finally, the new global model $M_{t+1}$ is distributed to each client for further model training. Algorithm 1 provides the complete pseudocode.
**Algorithm 1.** The FedAvg for ACP Identification**Require:** The clients set C; The number of local epoch E; The local minibatch size set B and the learning rate η;**Server:**initialize $\omega_{0}$
**for** each communication round *t* = 1, 2, 3 … **do**    distributes $\omega_{t}$ to all clients    **for** each client i∈C **do in parallel**        $W_{t+1}^{i}\leftarrow ClientUpdate(\omega_{t})$
    **end for**    average the model parameters    compute global model $\omega_{t+1}=\sum_{i=1}^{C}\frac{n_{i}}{n}W_{t+1}^{i}$
    Load the new model $\omega_{t+1}$ and get the new global model $M_{t}$
**end for****ClientUpdate(**$\omega_{t}$**):****for** each local epoch e from 1 to E **do**    **for** batch b∈B **do**        calculate loss and gradients $\nabla W_{t,b}^{k}$
        $W_{t+1}^{i}\leftarrow W_{t}^{i}-\eta \nabla W_{t,b}^{k}$
    **end for**
**end for****return**ω to server

Figure 1 illustrates the process of FL, which consists of several steps achieved by setting the corresponding communication rounds: global model distribution, client-server model training, client model parameter upload, and model parameter aggregation to obtain a new global model. The number of training epochs for each client model is set to 1. Experimental results demonstrate that using a smaller number of training rounds for this model can reduce overfitting and improve the model’s generalization capability.

### 3.4. Network Output Structure

After the training of the pre-trained model, sentence-level feature vectors are obtained. The first fully connected layer is utilized to capture the relationship between the input and the classes. In the last fully connected layer, the input text sentence’s probability of being an access control policy statement is computed based on the output feature y, using Softmax or Logsoftmax to calculate the data’s probability distribution. The calculations are shown in Equations (12) and (13).

(12) $$Soft\max_{p}(y)=\frac{\exp(y)}{\sum_{p}\exp(y)}$$

(13) $$Logsoft\max_{p}(y)=\log[\frac{\exp(y)}{\sum_{p}\exp(y)}]$$

where P represents the statement class (1 for ACP statement, 0 for non-ACP statement), and $Soft\max_{p}(y)$ and $Logsoft\max_{p}(y)$ denote the corresponding probabilities of the statement classes.

### 3.5. Analysis of the Model

The proposed model in this paper first utilizes a pre-trained language model to transform access control policies into word vector representations, capturing rich contextual information. Subsequently, the application of BiLSTM enhances the model’s understanding of long-term dependencies within sequential data, and the combination of both components allows for the fusion of multi-level information, thus improving the model’s comprehension of access control policies. The introduction of federated learning adds an additional layer of privacy protection, making the proposed approach suitable for applications requiring cross-organizational collaboration while safeguarding sensitive information. In conclusion, a flexible, interpretable, and privacy-conscious model is constructed, enhancing its effectiveness in addressing challenges related to access control policy recognition.

## 4. Experimental Results

In the experiments, we trained access control statement recognition models based on Bert-BiLSTM and Bert-CNN using centralized learning. Additionally, we trained the FL-Bert-BiLSTM model using distributed learning. Finally, we analyzed and compared the performance of these models using metrics, such as prediction accuracy, recall rate, and F1-score.

### 4.1. Data Source

ACP is widely used in various domains, including electronic healthcare, education, and conference management, among others. In this study, we evaluate the proposed method using publicly available multi-domain datasets as displayed in Table 2, namely, iTrust, IBM APP, Cyberchair, and Collected ACP datasets. These datasets were manually annotated by Slankas et al. [21]. The iTrust dataset is an open-source healthcare application; the IBM APP dataset is a course management system used in IBM universities; the Cyberchair dataset is related to conference management systems; and the Collected ACP dataset is a combination of ACP collected by Xiao et al. [20]. To overcome the limitation of having a small amount of data in a single dataset for centralized learning, we combine these four datasets for experimentation. The combined dataset contains 2477 instances, with 80% used for training and 20% used for testing.

### 4.2. Evaluation Metrics

As evaluation metrics, this study uses accuracy, precision, recall, and F1-score to assess the experimental performance. The parameters and calculation methods used in the evaluation are as follows: $T_{p}$ (True Positive) represents the number of ACP statements correctly identified, $T_{N}$ (True Negative) represents the number of non-ACP statements correctly identified, $F_{P}$ (False Positive) represents the number of ACP statements incorrectly identified, and $F_{N}$ (False Negative) represents the number of non-ACP statements incorrectly identified. Accuracy is the most commonly used and intuitive performance metric, representing the proportion of correctly identified ACP statements to the total number of statements. The calculation formula is given by Equation (14).

(14) $$\mathrm{Accuracy}=\frac{T_{P}+T_{N}}{T_{P}+T_{N}+F_{P}+F_{N}}$$

Precision is defined as the ratio of the number of correctly identified ACP statements to the total number of statements identified as ACP. The calculation formula is shown as Equation (15):

(15) $$\mathrm{Precision}=\frac{T_{P}}{T_{P}+F_{P}}$$

Recall is defined as the ratio of the number of correctly identified ACP statements to the total number of actual ACP statements. The calculation formula is given as Equation (16):

(16) $$\mathrm{Recall}=\frac{T_{P}}{T_{P}+F_{N}}$$

To provide a concise representation of model performance, F1-score is used, which is the weighted harmonic mean of Precision and Recall. A higher F1-score indicates higher values for both Precision and Recall, indicating a larger number of correctly identified ACP statements. The calculation formula is given as Equation (17):

(17) $$F1-\mathrm{score}=\frac{2\mathrm{Precision}\cdot \mathrm{Recall}}{\left(\mathrm{Precision}+\mathrm{Recall}\right)}$$

The software and hardware environment for the experiments in this study is as follows: the operating system is Ubuntu 18.04, the CPU is Intel Core i9-10900K@ 3.70 GHz, the GPU is GeForce RTX 3090, the memory is 32 GB, the PyTorch version is 1.10.2, the Numpy version is 1.23.3, the Transformers version is 3.02, and the Python version is 3.8.

### 4.3. Hyperparameter Settings

The hyperparameter settings for this experiment are as follows: The input layer utilizes the BERT pre-trained language model to convert access control policies into word vectors. Following the input layer, a BiLSTM layer is employed with hidden units set to 256, effectively leveraging both forward and backward feature information. A dropout layer with a rate of 0.1 is applied to enhance the model’s generalization ability. Finally, the model passes through two fully connected layers, utilizing the ReLU activation function and the LogSoftmax function, respectively. Model weights are saved and uploaded, and a federated aggregation algorithm is employed to obtain the new weight model. During the training process, the learning rate is set to 1 × 10^−5^, and the Adam optimizer is used for model training.

### 4.4. CL-Bert-BiLSTM vs. CL-Bert-CNN vs. CL-Bert-FC

Before training, the ACP statements are mapped to word vectors using the Bert model as input. Then, in the downstream models of BiLSTM, CNN, and fully connected (FC) networks, Adam optimizer and Cross-Entropy loss function are used. This results in the CL-Bert-BiLSTM, CL-Bert-CNN, and CL-Bert-FC approaches. The hyperparameters of the algorithm framework are adjusted to obtain better network performance.

**Figure 5:**
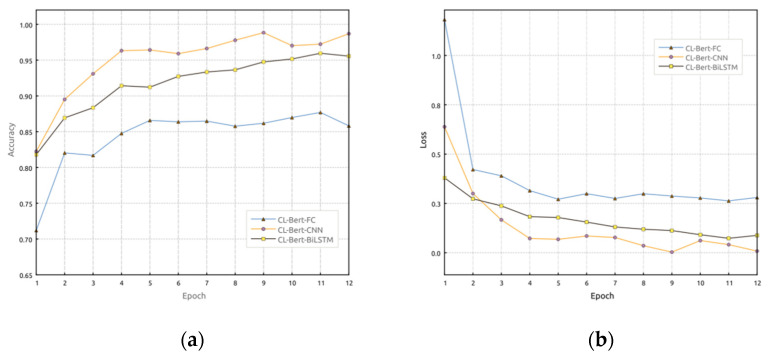
Performance comparison between the Bert-BiLSTM model, Bert-CNN model, and Bert-FC model: (**a**) accuracy comparison and (**b**) loss performance comparison.

During the training phase, the datasets from all clients are integrated into a centralized dataset for centralized training. Figure 5 illustrates the accuracy and loss rate results after 12 epochs of training in the centralized model. From Figure 5, it can be observed that after pre-training word embeddings using Bert, using CNN and BiLSTM as downstream models performs better in terms of accuracy, precision, F1-score, and other metrics in ACP statement recognition. The overall performance of the FC model, on the other hand, is not as good as that of the CNN and BiLSTM models.

### 4.5. FL-Bert-BiLSTM vs. FL-Bert-CNN vs. FL-Bert-FC

**Figure 6:**
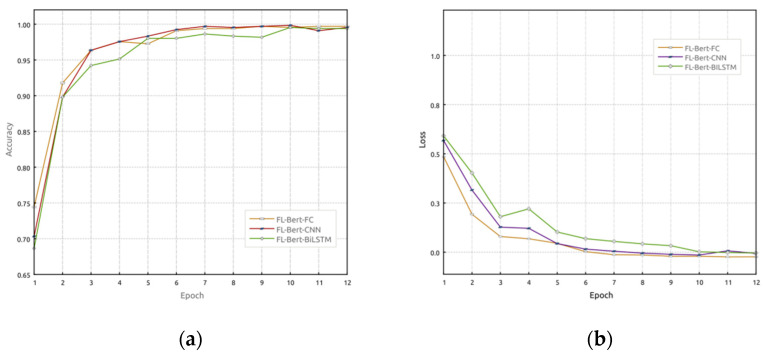
Performance comparison between FL-Bert-BiLSTM model, FL-Bert-CNN model, and FL-Bert-FC model: (**a**) accuracy comparison and (**b**) loss performance comparison.

In this section, the performance of the proposed FL-Bert-BiLSTM model is evaluated. Federated learning is employed, and a comparison is made among FL-Bert-BiLSTM, FL-Bert-CNN, and FL-Bert-FC. In this experiment, multiple users participate in the model training simulation. After four communication rounds, three clients are selected as participants, and the dataset is randomly divided into three parts and sent to each client for local model training. Figure 6 illustrates the accuracy and loss rate variations of FL-Bert-BiLSTM, FL-Bert-CNN, and FL-Bert-FC models over 12 epochs of training. From Figure 6, it can be observed that during the training process, updating the global model through the central server’s aggregation in each round allows the retention of the client’s training features. The accuracy shows an increasing trend, while the loss rate continuously decreases. Among them, FL-Bert-BiLSTM outperforms the other models in terms of performance.

### 4.6. Federated Learning (FL) vs. Central Learning (CL)

**Figure 7:**
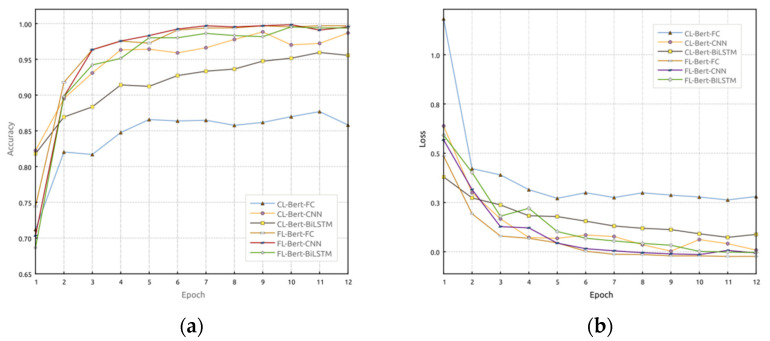
Performance comparison between FL and CL: (**a**) accuracy comparison and (**b**) loss performance comparison.

Figure 7 demonstrates the performance comparison between FL and CL across different models. It is evident that under the federated learning approach, the accuracy of each model surpasses that of centralized learning. Additionally, the model loss rate is lower, accelerating the convergence speed and improving the model’s generalization ability. The training results for Figure 5, Figure 6 and Figure 7 are given in Table 3, and the test results are given in Table 4, where FL-Bert-BiLSTM achieves an accuracy of 94.12% and an F1-score of 93.07%. In comparison with other models, significant improvements are observed in terms of accuracy and F1-score. Therefore, the proposed FL-Bert-BiLSTM model exhibits significant performance enhancement compared to CL-Bert-BiLSTM, enabling accurate identification of access control policy statements.

## 5. Discussion

The primary objective of this research is to achieve privacy-preserving access control policy recognition for sensitive data in the context of IoT networks. In pursuit of this objective, we propose an IID (Independently Identically Distributed) method based on FL-Bert-BiLSTM, which combines federated learning with pre-trained language models and deep learning techniques. Our method aims to strike a balance between protecting user data privacy and ensuring the accuracy of policy recognition, aligning with the core principles of IoT networks’ edge computing and AI applications.

The experimental results validate the effectiveness of our proposed method. By incorporating contextual features through the Bert-BiLSTM model, we observed a substantial improvement in the accuracy of access control policy recognition. The Bert-BiLSTM model provides richer semantic information, enhancing the understanding and classification of policies, which is of utmost importance in accurately recognizing access control policies, especially in complex and dynamic IoT scenarios with diverse devices at the edge.

To address privacy concerns in the context of IoT networks, our FL-Bert-BiLSTM model leverages federated learning techniques. By performing weighted averaging of models trained by individual clients, we ensure that user privacy requirements are met while achieving good recognition performance. The use of federated learning allows us to collaboratively train the model without compromising the privacy of individual client data, making it well-suited for edge computing environments, where data security and privacy are critical considerations.

However, federated learning may potentially introduce some additional energy consumption concerns for IoT devices with limited power resources. This depends on specific scenarios and implementations. A key factor is the selection and size of the model. Larger models require more storage and computational resources, potentially exerting pressure on devices with limited power budgets. To address this situation, considerations may include the adoption of lightweight models such as MobileBert, Bort, or model pruning techniques to reduce model parameters and associated computational requirements. Alternatively, model quantization techniques can represent model parameters as low-precision values, thereby reducing storage and computational demands.

In federated learning, devices need to participate in model updates and parameter transmission, which may lead to additional energy consumption. For IoT devices with limited power resources, this could shorten device battery life. To mitigate energy consumption, optimizing communication protocols, reducing communication frequency, adopting more efficient federated learning algorithms, or designing low-power hardware at the hardware level and adjusting algorithms and models accordingly could be considered. These measures can assist IoT devices in participating more effectively in federated learning.

In the future, our research aims to further enhance the accuracy of policy recognition in a federated learning environment tailored for IoT networks. We plan to explore the integration of additional features into the access control policy recognition system, such as temporal information or user behavior patterns, to capture the dynamics of IoT networks accurately. By incorporating these features, we expect to improve the overall performance and robustness of the system, enabling effective policy recognition in real-time IoT applications.

This research makes a significant contribution to the field of access control in the context of IoT networks. The IID approach based on FL-Bert-BiLSTM addresses the inherent challenges of preserving user data privacy and ensuring accurate policy recognition within dynamic IoT environments. The experimental results and insights obtained from this study provide valuable directions for the further development of policy recognition technology in real-world IoT scenarios. Future research efforts will focus on refining the system and exploring additional features to enhance the accuracy and applicability of access control policy recognition in real-world IoT environments.

## 6. Conclusions

In this study, we present an innovative approach for access control policy recognition that leverages a combination of pre-trained language models, deep learning models, and federated learning algorithms. The primary focus of our research is to achieve privacy-preserving policy recognition in the context of IoT networks, by effectively addressing the challenges of limited labeled data and data privacy concerns.

Our proposed FL-Bert-BiLSTM framework demonstrates promising results in accurately recognizing access control policies while ensuring data privacy and security. By incorporating pre-trained word embeddings and leveraging federated learning, our approach achieves a balance between accuracy and privacy protection. The Bert-BiLSTM model captures rich semantic information from policy documents, enhancing the understanding and classification of policies, which is crucial in complex IoT environments with diverse edge devices.

The integration of federated learning in our approach enables collaborative training using data from multiple clients, avoiding the need for centralized data collection. This decentralized model training process aligns well with the principles of IoT networks, where data are processed closer to the source, reducing communication overhead and enhancing real-time decision-making in IoT networks.

Our research contributes to the intersection of IoT networks and artificial intelligence in the context of access control policy recognition. The proposed FL-Bert-BiLSTM framework offers an effective and privacy-aware solution for securing IoT networks and edge devices, where data privacy and accuracy are of paramount importance.

Looking ahead, we envision further exploring the potential of integrating additional AI techniques and edge computing paradigms to improve the scalability and adaptability of our approach in diverse IoT network scenarios. The evolution of edge computing and AI will undoubtedly present new challenges and opportunities for enhancing security and intelligence in IoT systems.

## Figures and Tables

**Table 2:** Dataset information.

Dataset	Domains	ACP Statements	Non-ACP Statements	Total
iTrust	Healthcare	967	664	1631
IBM App	Education	169	232	401
Cyberchair	Conference	140	163	303
Collected ACP	Multiple	125	17	142
Total	—	1401	1076	2477

**Table 3:** Training result performance of all methods.

	Algorithm	CL-Bert-FC		CL-Bert-CNN		CL-Bert-BiLSTM		FL-Bert-FC		FL-Bert-CNN		FL-Bert-BiLSTM	
Epoch		Accuracy	Loss	Accuracy	Loss	Accuracy	Loss	Accuracy	Loss	Accuracy	Loss	Accuracy	Loss
Ep.1		71.19%	1.231	82.24%	0.680	81.79%	0.418	74.43%	0.525	70.32%	0.609	68.64%	0.632
Ep.2		82.04%	0.460	89.51%	0.337	86.93%	0.310	91.78%	0.229	89.80%	0.353	89.80%	0.441
Ep.3		81.69%	0.429	93.09%	0.202	88.35%	0.274	96.34%	0.114	96.35%	0.162	94.21%	0.216
Ep.4		84.76%	0.352	96.32%	0.107	91.42%	0.219	97.56%	0.103	97.56%	0.156	95.13%	0.256
Ep.5		86.58%	0.308	96.42%	0.102	91.22%	0.214	97.26%	0.079	98.33%	0.077	98.02%	0.137
Ep.6		86.38%	0.337	95.91%	0.119	92.73%	0.191	99.08%	0.035	99.23%	0.049	98.02%	0.103
Ep.7		86.48%	0.312	96.62%	0.112	93.34%	0.166	99.39%	0.020	99.69%	0.038	98.63%	0.089
Ep.8		85.77%	0.336	97.78%	0.069	93.64%	0.154	99.39%	0.019	99.54%	0.028	98.33%	0.076
Ep.9		86.18%	0.325	98.84%	0.036	94.75%	0.147	99.69%	0.012	99.69%	0.022	98.17%	0.068
Ep.10		86.98%	0.315	97.02%	0.096	95.16%	0.126	99.54%	0.012	99.84%	0.018	99.54%	0.035
Ep.11		87.69%	0.300	97.23%	0.075	95.96%	0.107	99.69%	0.008	99.08%	0.040	99.39%	0.031
Ep.12		85.82%	0.317	98.69%	0.041	95.56%	0.120	99.70%	0.009	99.54%	0.026	99.39%	0.029

**Table 4:** The performance of all methods.

Algorithm	Accuracy	Recall	Precision	*F*_1_-Score
CL-Bert-FC	89.72%	90.50%	88.23%	89.01%
CL-Bert-CNN	92.34%	93.19%	91.20%	91.99%
CL-Bert-BiLSTM	92.74%	91.62%	92.44%	92%
FL-Bert-FC	93.51%	89.25%	95.50%	92.27%
FL-Bert-CNN	93.71%	89.25%	95.98%	92.49%
FL-Bert-BiLSTM	94.12%	91.12%	95.12%	93.07%
